# Complete genome sequence of *Enterobacter cloacae* ST462 harboring the *mcr-9* gene on the chromosome, isolated from a pediatric diarrhea patient in Southern Vietnam

**DOI:** 10.1128/mra.01176-24

**Published:** 2025-01-27

**Authors:** Nga Thi Le, Phuong Hoai Hoang, Chinh Van Dang, Tinh Huu Ho, Phuc Le Hoang, Dinh Quang Truong, Ha Thi Thanh Nguyen, Chuong Van Le, Minh Nhat Ha Truong, Tuom Thi Tinh Truong, Trang Thi Phuong Phan

**Affiliations:** 1 Center for Bioscience and Biotechnology, University of Science, Ho Chi Minh, Vietnam; 2 Vietnam National University, Ho Chi Minh, Vietnam; 3 Institute of Public Health, Ho Chi Minh, Vietnam; 4 Children’s Hospital No. 1, Ho Chi Minh, Vietnam; 5 City Children’s Hospital, Ho Chi Minh, Vietnam; 6 University of Medicine and Pharmacy, Ho Chi Minh, Vietnam; University of Maryland School of Medicine, Baltimore, Maryland, USA

**Keywords:** *mcr-9.1*, *mcr-9*, colistin

## Abstract

Colistin resistance threatens global health as it compromises the effectiveness of a last-resort antibiotic. We present the complete genome sequence of *Enterobacter cloacae* ST462, which carries the *mcr-9.1* gene, isolated from a pediatric diarrhea case in southern Vietnam. The 5,049,362 bp genome contains 24 resistance genes distributed across 107 contigs.

## ANNOUNCEMENT

Colistin resistance, associated with the mobile *mcr-9* gene, poses a significant global public health threat, particularly in the treatment of multidrug-resistant Gram-negative infections (1). This study reports the complete genome sequence of colistin-resistant *Enterobacter cloacae* ST462 (NCE259), isolated from a pediatric diarrhea patient in southern Vietnam. The genome harbors the *mcr-9.1* gene and 23 other antibiotic resistance genes, offering valuable insights into colistin resistance mechanisms to aid global antimicrobial resistance surveillance.

The NCE259 strain was isolated from a rectal swab collected from a pediatric patient (aged 0–5 years) at Children’s Hospital No. 1, Ho Chi Minh City, Vietnam. Samples were cultured on Violet Red Bile Glucose Agar (Merck, Germany) at 37°C for 24 hours. Pink and purple colonies were selected and purified on nutrient agar. Single colonies were used for antimicrobial susceptibility testing and DNA extraction. Strain NCE259 was resistant to colistin (minimum inhibitory concentration, [MIC] 4 mg/L), as determined using the Clinical and Laboratory Standards Institute, (CLSI) (2022) broth microdilution method. The presence of the *mcr-*9 gene was confirmed via PCR (2).

Genomic DNA was extracted from a colony cultured in tryptic soy agar broth (Merck, Germany) at 37°C for 16–18 hours under aerobic conditions. Whole-genome sequencing was performed on the DNBSEQ platform by Ktest. DNA libraries were prepared using the NEBNext Ultra II DNA Library Prep Kit (NEB, USA), following the manufacturer’s instructions. Paired-end sequencing with a read length of 150 bp (150PE) produced high-quality data with a genome coverage of 500×. Paired-end sequencing (150 bp) yielded 6,680,952 reads with 500× coverage, which was processed by Fastp v.0.23.1 (3) to filter data with Phred score <Q30).

The genome of *Enterobacter cloacae* ST462 NCE259 was *de novo* assembled using Unicycler v.0.4.8 (4), yielding 107 contigs with a total genome size of 5,049,362 bp and a GC content of 54.73%. The assembly was evaluated with QUAST v.5.2.0 (5), confirming high-quality metrics with 100% completeness and 0.4% contamination, as assessed by CheckM v.1.2.1 (6). Genome annotation, performed with Prokka v.1.14.6 (7), identified 4,717 coding sequences (CDS), 76 tRNAs, and 4 rRNAs. Resistance genes were detected using ABRicate v.1.0.1 with default minimum identity and coverage threshold (8), confirming the presence of the *mcr-9.1* gene responsible for colistin resistance, along with other resistance genes. The genome had an N50 value of 133,633 bp, providing robust data for further understanding resistance mechanisms. All results are summarized in Table 1.

**TABLE 1:** Genomic characteristics of *Enterobacter cloacae* ST462 carrying

Characteristic	NCE259
Assembly method	Unicycler v.0.4.8
Annotation method	Prokka v.1.14.6
Sequencing technology	DNBSEQ-G99
Genome coverage	500×
Contigs	107
Genome size (bp)	5,049,362
CDS	4,717
No. of tRNA	76
No. of rRNA	4
N50	133,633
L50	11
GC content (%)	54.7
Resistance genes	*mcr-9.1, ampC, crp, tolC, uppP, cpxA, marA, bepF, oqxB, mdtB, mdtC, baeR, acrA, acrB, dhfrI, tetA, qnrS1*, *fosA, emrB, emrA, mprA, folP, acrB, msbA*

Bacterial identification was conducted using GTDB-Tk v.2.1.1 (9, 10), confirming the strain NCE259 to be *E. cloacae* with an average nucleotide identity of 98.4% compared to the reference genome of *E. cloacae* (GCF_000025565.1). The Multilocus Sequence Typing (MLST) analysis was performed using the mlst v.2.23.0 software and the PubMLST website (11),with alleles similarity threshold set at 95% sequence identity and 10% sequence coverage. The analysis classified the strain as ST462 based on seven housekeeping genes, with no novel mutations detected. Default parameters were used for all software unless otherwise specified.

## Data Availability

This complete genome sequence of NCE259 has been deposited in GenBank under accession no. JBEEIF000000000. The version described in this paper is the first version, JBEEIF000000000.1 (https://www.ncbi.nlm.nih.gov/Traces/wgs/JBEEIF01). The DNBSEQ sequence reads were deposited in the Sequence Read Archive (SRA) database under accession number PRJNA1091812.
